# A quick guide on implementing and quality assuring 3D printing in radiation oncology

**DOI:** 10.1002/acm2.14102

**Published:** 2023-07-27

**Authors:** Michael Ashenafi, Seungkyo Jeong, Joshua N. Wancura, Lang Gou, Matthew J. Webster, Dandan Zheng

**Affiliations:** ^1^ Department of Radiation Oncology University of Rochester Medical Center Rochester New York USA; ^2^ Department of Applied Mathematics University of Rochester Rochester New York USA

**Keywords:** 3D printing, additive manufacturing, bolus, quality assurance, radiation oncology, skin brachytherapy

## Abstract

As three‐dimensional (3D) printing becomes increasingly common in radiation oncology, proper implementation, usage, and ongoing quality assurance (QA) are essential. While there have been many reports on various clinical investigations and several review articles, there is a lack of literature on the general considerations of implementing 3D printing in radiation oncology departments, including comprehensive process establishment and proper ongoing QA. This review aims to guide radiation oncology departments in effectively using 3D printing technology for routine clinical applications and future developments. We attempt to provide recommendations on 3D printing equipment, software, workflow, and QA, based on existing literature and our experience. Specifically, we focus on three main applications: patient‐specific bolus, high‐dose‐rate (HDR) surface brachytherapy applicators, and phantoms. Additionally, cost considerations are briefly discussed. This review focuses on point‐of‐care (POC) printing in house, and briefly touches on outsourcing printing via mail‐order services.

## 1 INTRODUCTION

3D printing, or additive manufacturing (AM), has become a widespread technology in medical fields with numerous applications. In radiation oncology, many studies have investigated the clinical implementation and development of 3D printed devices, validating the application of 3D printing and demonstrating superiority to conventional devices in many aspects.^1^, ^2^, ^3^, ^4^, ^5^, ^6^, ^7^ A wide range of clinical applications have been reported such as patient‐specific bolus, immobilization, and shielding devices, various high‐dose‐rate (HDR) brachytherapy applicators and surgical or insertion guides, customized phantoms for quality assurance (QA) and research, and other random devices.^8^, ^9^, ^10^, ^11^ In this paper, we chose to focus on three popular clinical applications of 3D printing in radiation oncology: patient‐specific bolus, HDR skin/surface brachytherapy applicators, and customized phantoms.

Tissue‐equivalent boluses are often used to improve the quality of radiotherapy treatments by increasing dose to superficial targets in photon and electron external beam therapy. However, a common issue with commercially available boluses of uniform thickness is that they may fail to make perfect contact with the patient's skin, especially where the surface is irregular. This can result in substantial air gaps that distort the desired dosimetric effect. 3D‐printed boluses have been shown to significantly reduce the amount of air gap and to improve dosimetric accuracy,^12^, ^13^, ^14^, ^15^, ^16^, ^17^ and have also been applied to offer modulated electron therapy.^18^, ^19^, ^20^

Similarly, commercially available brachytherapy skin applicators have had difficulties treating regions with irregular surface contours due to their inflexibility in catheter configurations. Fitting these applicators to the skin surface in a proper and consistently reproducible manner requires well‐trained staff and highly developed manual skills. 3D‐printed HDR skin applicators have been shown to achieve better conformity and dose distributions while improving reproducibility.^4^, ^5^, ^21^, ^22^, ^23^

The demand for customized phantoms for QA and research has increased with the advancement of radiotherapy technologies, which are now capable of delivering highly complex and personalized treatment plans. Conventionally, there has been a limited selection of these phantoms commercially available, and custom‐made phantoms from machine shops usually have relatively simple designs. With the advent of and increasing access to 3D printing, more complex customized phantoms have become popular. Phantoms as complex as anthropomorphic phantoms can be made by 3D printing. Several studies have demonstrated that modification of printing parameters and materials can simulate different tissue types, achieve heterogeneity in customized anthropomorphic phantoms, and create variable density phantoms.^6^, ^24^, ^25^, ^26^, ^27^ Researchers and clinicians can therefore more conveniently conduct versatile experiments with their customized phantoms enabled by 3D printing.

Thanks to substantially improved cost and accuracy over the past decade, 3D printing has become increasingly common in radiation oncology departments for routine clinical applications and new clinical developments. For these and future new users in radiation oncology, there is a need for more guidance on the proper implementation, use, and ongoing QA of the 3D printing technology and workflows. In this paper, we will attempt to discuss and make recommendations on workflow instructions and quality assurance for 3D printing, focusing on the above three main categories of applications in radiation oncology, based on existing literature and our own experience. Cost considerations will also be briefly discussed. There are always more applications being invented, and these guidelines will need to be adapted to any given clinic's specific circumstances and use cases. Also, our paper focuses on point‐of‐care (POC) printing in house, whereas the increasingly popular outsourcing mail‐order 3D printing services will be briefly discussed.

## 2 3D PRINTING EQUIPMENT AND SOFTWARE

### 2.1 3D printer

The first step of an in‐house 3D printing implementation is to choose the right 3D printer. Currently, a 3D printer can be purchased at a few hundred to several thousand dollars, depending on technology and performance.

In terms of technology, there are three main types of 3D printers: stereolithography (SLA) printers,^28^, ^29^ selective laser sintering (SLS) printers,^30^, ^31^ and fused deposition modeling (FDM) printers.^32^, ^33^ In SLA, or vat photopolymerization (VPP) according to the ISO/ASTM 52 900 nomenclature, layer‐by‐layer printing is achieved by fusing liquid resins with a reflected laser. This technique achieves high spatial resolution on the order of tens of micrometers, but the printing materials can be costly and the printing speed is slow. In SLS, or powder bed fusion (PBF), a laser sweeps across a powered material, fusing the particles on the surface to achieve layer‐by‐layer printing in an enclosed chamber. SLS printing resolution is slightly lower than SLA, but higher than FDM, or material extrusion (MEX). SLS has the highest printing speed among the three, and also the highest printer cost. An FDM printer uses a robotic extrusion nozzle with sizes usually on the order of sub‐millimeter to melt and fuse filaments to form 3D objects, also in a layer‐by‐layer fashion. Common FDM filaments have relatively lower costs, compared to SLA. However, the printing resolution is lower and needs to be balanced against the printing time in choosing nozzle size. Of the three printer types, FDM printers seem to be most common from studies that reported in‐house 3D printing applications in radiation oncology,^1^, ^2^, ^12^, ^14^, ^23^, ^25^, ^26^, ^32^, ^34^, ^35^, ^36^, ^37^, ^38^, ^39^, ^40^, ^41^, ^42^, ^43^, ^44^ possibly due to the lower installation and operational costs. On the other hand, vendors that provide mail‐order services for 3D‐printed devices to radiation oncology departments often offer SLS‐ or SLA‐printed devices, in addition to FDM‐printed ones, for versatile materials and finishes of the products.

Both initial and operational costs should be considered when choosing the right 3D printer. Printing capacity needs to be considered based on the maximum size of prints needed. The post‐processing functions and speed should also be considered, and a work area will need to be selected to house the printer. Factors to consider for this include manual post‐processing, noise, and environmental safety. Often, filing and sanding of printed objects with Dremel tools may require a workspace and proper containment of debris. Proper ventilation may also be needed for certain printing materials.

### 2.2 Software and data conversion steps

**FIGURE 1:**
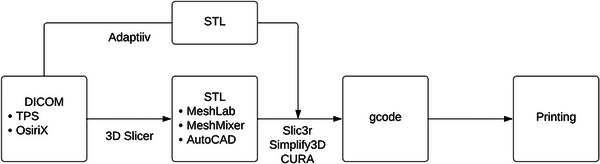
Main steps of a 3D printing workflow demonstrating file formats and example software.

For the three main 3D printing applications in radiation oncology discussed in this paper, the data conversion step shares the same basis: start from DICOM, convert into STL, then into gcode for printing commands. Figure 1 depicts a schematic of these major steps. In the following paragraphs common software will be discussed for these steps, together with some cost and webpage information that was collected at the time of writing and subject to future changes by the vendor or developer. Table 1 also summarizes the software information.

**TABLE 1:** A list of commonly used software in radiation oncology 3D printing workflow.

Name	Use	Cost	Note
Eclipse, RayStation, Pinnacle, Monaco, etc.	Treatment planning (registration, delineation, initial contouring, etc.)	–	
OsiriX	DICOM analysis; generally used in addition to TPS	$70+/month	Only supports MacOS
3D Slicer	Multipurpose for visualization, processing, segmentation, registration, analysis; Conversion from DICOM to STL	Open source	Has a useful forum for developers and users
MeshLab	3D mesh processing	Open source	
MeshMixer	3D mesh processing	Open source	
AutoCAD	3D mesh processing	$160+/month, but may vary	
Fusion 360	3D mesh processing	$70/month	
Polyworks	3D mesh processing	Need to contact the vendor for quotes	
Slic3r	Conversion from STL to gcode	Open source	
Simplify3D	Conversion from STL to gcode	$149/month, but may vary	
Ultimaker CURA	Conversion from STL to gcode	Open source	Preferred for Ultimaker printers, but may be used for others as well
Adaptiiv	Multipurpose for 3D printed bolus and surface brachytherapy applicator; conversion from DICOM to STL, cropping, smoothing, in vivo dosimetry, channel positioning and numbering	Need to contact the vendor for quotes	Combines multiple software into one; tailored to radiation oncology

For these printed devices, the design usually comes from CT images, but can also be derived from other imaging modalities such as 3D surface scans and magnetic resonance imaging (MRI), or may alternatively be created directly by users. The images are usually viewed on a treatment planning system (TPS). For anthropomorphic phantoms, other DICOM software with advanced post‐processing and analysis functions such as OsiriX may be used in addition to TPS, or even replace, for segmentation and contouring. OsiriX can cost $70+ per month. The processed DICOM is then converted into STL using software such as 3D Slicer, a free, and open‐source image analysis software.^45^ Note that even though most studies seem to utilize 3D Slicer as an image file converter, it can be used for other purposes as well, as it provides a variety of functionalities, including processing, registration, visualization, automatic image segmentation, and analysis of medical 3D images and meshes. For additional information, one may refer to the software documentation which is available on their website (slicer.org).

Once converted, the STL files are imported into a 3D mesh processing software such as MeshLab (meshlab.com), MeshMixer (meshmixer.com), or AutoCAD (Autodesk.com) for smoothing and additional editing to improve seamless contact between the device and patient. MeshLab and MeshMixer are free and open‐source while AutoCAD can cost $160+ per month. Once the editing is done, the mesh is converted into gcode for printing, using programs such as Slic3r, Simplify3D, or Ultimaker CURA. CURA and Slic3r can be downloaded free while Simplify3D costs $149.

Adaptiiv (adaptiiv.com), a comprehensive radiotherapy‐specific 3D printing software, can be an attractive alternative to the list of programs above if one wishes to have a one‐stop shop. It supports post‐processing features for both bolus and HDR surface brachytherapy applicators, including image processing (DICOM to STL), cropping, smoothing, in vivo dosimetry, channel positioning, editing, numbering, etc.

### 2.3 Materials and printing parameters

Numerous factors such as tissue equivalency, flexibility, printability, biocompatibility, antibacterial properties, and cost must be taken into account during material selection.^14^

FDM printers are popular in radiation oncology departments. Polylactic acid (PLA) and acrylonitrile butadiene styrene (ABS) are the two most widely used materials with FDM printers for 3D printed boluses, applicators, and water‐equivalent phantoms. Both materials have water‐like densities at 1.0–1.2 g/cm^3^ and are widely available at reasonable costs of $5 per 10‐meter filament spool. Therefore, they have been used in numerous studies and shown to have satisfactory dosimetry with ionization chamber and other dose measurements.^2^, ^25^, ^26^, ^34^, ^46^ PLA is made of natural sugars and is more biodegradable than most other plastics, but it is also more brittle. ABS can also be recycled fairly easily and is a more resilient material, but it tends to shrink at cooling and emits a fume when printing that requires better ventilation. Variations of standard ABS have also been used, such as the tougher ABS M‐30, and ABSplus which reduces shrinkage while increasing hardness.^13^, ^47^

While PLA and ABS have been widely applied as boluses and applicators in radiation oncology, both are fairly rigid and stiff, which can be uncomfortable for patients, especially if the skin is already inflamed. Recent studies have investigated alternative materials for 3D‐printed boluses, such as Clear Flex 30, Agilus‐60, and hydrogel.^4^, ^7^, ^16^, ^40^ These materials demonstrated improved flexibility and satisfactory dosimetry. However, they were also found to cause skin irritation in some patients and suffered from high production cost, poor mechanical properties, and a complicated production process. Another study compared three materials for 3D boluses, Clear Flex 30, NinjaFlex, and Dragon Skin, recommending Dragon Skin out of the three based on lower cytotoxicity and air gap.^16^ Other studies also showed that various silicone materials are pliable, conform well to irregular surfaces, and are free of cytotoxicity and skin irritation.^16^, ^17^, ^48^ Another semi‐flexible material, theroplastic polyurethane (TPU), was shown to be an effective and practical bolus choice for radiotherapy.^15^ Elastomer materials such as TPU and TangoPlus were shown to be adequate alternatives to PLA and ABS, as they are able to provide excellent physical and chemical properties, including flexibility, biocompatibility, and elasticity, without major downside.^14^ Another increasingly popular approach to make more biocompatible 3D bolus is to 3D print a mold and then pour a silicone mix in to create the 3D bolus.^16^, ^49^ In addition to more versatile material choices, this approach could also reduce print time and cost. The search for the optimal material with good dosimetry, flexibility, biocompatibility, and cost‐effectiveness is still ongoing.

With different printers, printing materials, and applications, printing parameters need to be optimized for the task. Printing temperature selection is straightforward and is usually based on the printing material. For example, due to the lower glass transition temperature of 65°C for PLA, the temperature at which it becomes malleable, the nozzle temperature is set lower for PLA than for ABS in 3D printing. For PLA, it is usually set around 180°C, while ABS requires above 200°C. Moreover, due to shrinkage issues, the print bed must be set higher for ABS devices at around 100°C, whereas 50−60°C is sufficient for PLA. More research on optimal printing parameters may be warranted, as only a few studies have explored the effects of printer parameters on the final performance of the printed devices.^34^, ^35^, ^40^ It is worth noting that universally optimal print parameters probably do not exist. Instead, they are highly dependent on an individual printer and the specific use task.

The choice of nozzle diameter requires a compromise between resolution and printing time. It is usually set between 0.4 and 0.6 mm.^4^, ^17^, ^26^, ^42^ However, one of these studies also successfully created a variable density phantom using 0.8 mm diameter, showing that there is no significant loss of detail as long as the diameter is set to be smaller than the CT pixel size, which is ∼1 mm in most cases.^26^ This would help reduce the printing time and cost.

A few studies have evaluated the dosimetric impact of different infill percentages for 3D boluses,^35^, ^40^, ^44^ finding that higher infill percentage corresponds to higher density and homogeneity within the bolus material, which indicates better dosimetry. It was also reported that bolus density and homogeneity not only depend on infill percentage but also on infill pattern and even printing direction. One study investigated the effects of printing parameters for PLA boluses. They reported that a sunglass infill pattern, 60% infill, and horizontal printing direction are the optimal parameter settings for 6MV photon beam therapy.^35^ A similar study evaluated these printing parameters for 3D printed immobilization devices.^34^ Among the 10 studied infill patterns, the stars infill pattern was found to best balance between surface dose and device strength, while a dependence on printing orientation was also noted. Another study reported that a 20% infill density change in ABS printing has no significant effect on dose distribution for 3D‐printed HDR applicators, unlike for 3D‐printed boluses.^41^ They therefore recommended that HDR applicators could be printed at low infill percentages with ABS on a low‐cost printer to reduce the production time and cost. In our in‐house study of 3D printed HDR skin applicators with PLA, decreasing the infill density from the default 95% to 90% resulted in acceptable dosimetric dosimetry of ∼2%. At the same time, this only led to 3% printing material and 2% printing time saving. Further reduction to 50%−85% infill yielded dosimetric differences between 27% and 11%, and infill percentage below 50% could lead to dosimetric differences 30% or higher.

Clearly, more evaluations of printing parameter settings are needed for more printing materials, applications, and forms of treatment such as brachytherapy and external therapy with photon, electron, or proton beams.

## 3 WORKFLOW PROCEDURES

### 3.1 Fabrication of Boluses

**FIGURE 2:**
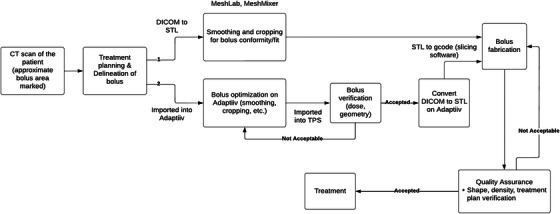
A typical workflow for direct 3D bolus fabrication using in house printing.

The typical workflow of directly printing a 3D bolus is depicted in Figure 2. The fabrication process begins with an imaging scan of the patient. Other imaging techniques may be used, but CT is the most widely used method for this purpose. The acquired images are then imported into a TPS, where the bolus and other regions of interest are delineated to establish a patient‐specific treatment plan. During this process, the thickness of the bolus is determined based on the tumor volume and the treatment plan. In this step, proper thresholding for body surface determination is important to avoid compression or air gap on the patient skin.^50^ The bolus design is then converted into STL using an image processing software and adjusted to enhance conformity to the patient using a 3D mesh processing software. Finally, the STL is converted to printing instruction format (gcode) using slicer software and sent for printing.

Alternatively, the bolus structure along with the CT images can be exported to radiation oncology specific software like Adaptiiv to further optimize the design. This software provides tools to smooth, crop, cleave, and modulate bolus thickness. Optimized bolus design is then imported back to the TPS for geometric and dosimetric verification. Once verified in the TPS, Adaptiiv software is used to covert bolus design from DICOM format into STL. Printer software is then used to convert from STL to gcode, and the bolus is printed out.

After the bolus has been printed and inspected, it may be used for treatment, though a re‐simulation of the patient with the printed device is often helpful.

In lieu of direct printing, another increasingly popular approach for 3D bolus fabrication is mold casting.^16^, ^49^ With this approach, a bolus‐shaped hollow mold is printed similar to the above‐described workflow. Support structures can be added in printing to the thin mold when necessary. The casting mold may also be printed in parts and later attached together to create bolus molds bigger than the printing capacity of the printer. After the bolus mold is fabricated, casting materials such as silicone mixes are poured into the mold and cured to create the bolus. Some advantages of this approach include the versatile material choices and reduced print time and cost.

**FIGURE 3:**
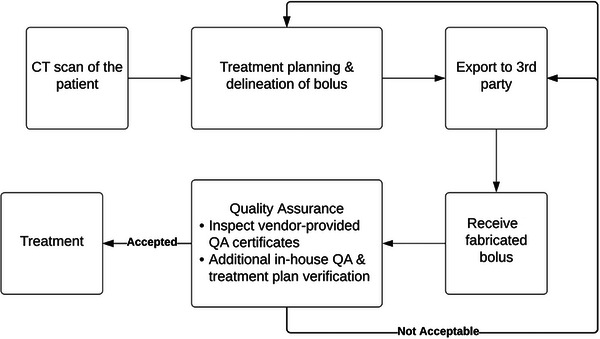
A typical workflow for 3D bolus fabrication via outsourcing printing to a third‐party mail‐order service.

While this paper focuses on POC printing in house, here we will use one example of bolus fabrication as shown in Figure 3 to illustrate an alternative outsourcing workflow to use mail‐order services from design software vendors or other third‐party customer device vendors. Vendors usually provide both directly‐printed 3D boluses and those using the mold casting. The third‐party 3D fabrication is gaining popularity in radiation oncology departments owing to the convenience, accessibility to versatile printing technologies and materials, etc.

### 3.2 Fabrication of HDR skin brachytherapy applicators

**FIGURE 4:**
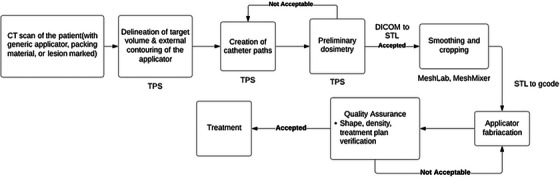
A typical workflow for 3D‐printed HDR surface brachytherapy applicator fabrication.

A typical workflow for 3D printing a HDR skin or surface brachytherapy applicator is depicted in Figure 4. Similar to the bolus workflow, CT images of the patient are acquired and imported into a TPS. Based on the CT images, the target volume is defined, and the external designing of the applicator is done. Subsequently, the ideal catheter paths in the applicator are created to efficiently cover the target volume and their curvatures are examined to avoid any problems regarding the catheter and channel, such as the catheter breaking inside the channel or excessive friction between the channel and catheter. This step could be done in a brachytherapy TPS.

**FIGURE 5:**
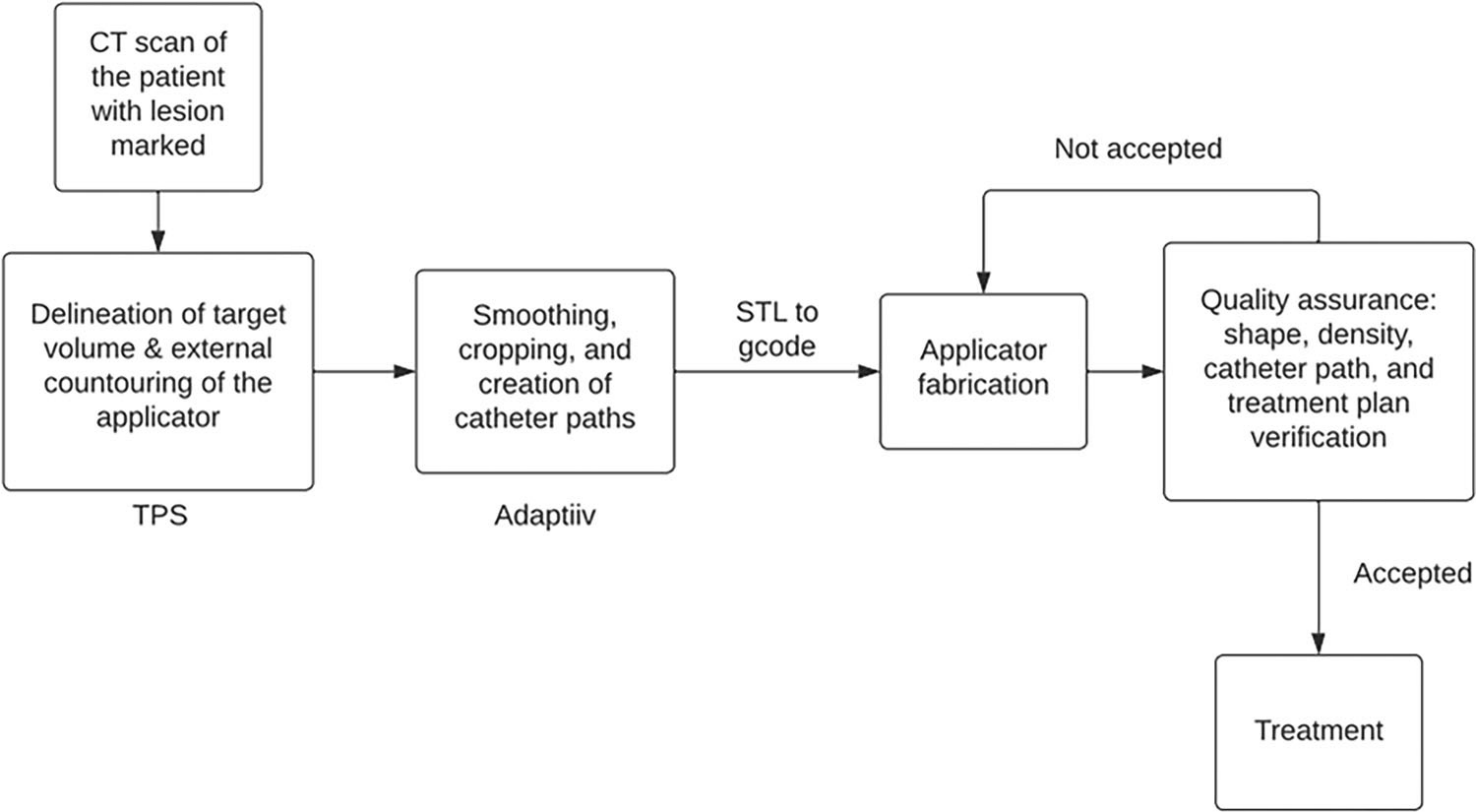
A workflow for 3D‐printed HDR surface brachytherapy applicator fabrication using Adaptiiv software.

Alternatively, as described above in Section 3.1, this can also be done using radiation oncology specific software like Adaptiiv. In this workflow as depicted in Figure 5, structure set and CT image data set are exported to Adaptiiv, in which a virtual applicator is created based on the target location and geometry. Depending on the catheter used and the afterloader type, Adaptiiv will automatically place catheter paths/channels inside the applicator to efficiently cover the target volume. Channel number, orientation, and curvature can be further optimized by the user to meet specific needs. The skin applicator is then printed following data conversion steps described above.

After the skin/surface applicator is printed and inspected, the patient is brought back and the applicator is securely positioned on the target area with catheters in place. Markers are then inserted inside catheters to easily identify the source path. Simulation CT images are acquired with the applicator in place and the image data are transferred to the TPS to generate a treatment plan. After the treatment plan is established according to the applicator design, the dose distribution is evaluated. If the distribution is not acceptable, the paths should be redesigned and reprinted following the data conversion steps described above.

In addition to skin/surface applicators, 3D printing has also been used for other HDR brachytherapy applications such as fabricating intracavitary applicators and insertion molds.^8^, ^10^ While their fabrication processes may share similarities with the skin application fabrication described here, there are also steps specific to a given use case and it might differ based on individual experience.

### 3.3 Fabrication of phantoms

Customized phantoms are fabricated using 3D printing for various QA and research purposes in radiation oncology. These phantoms conveniently facilitate machine QA, patient‐specific QA, and other development. Here we use anthropomorphic phantoms as an example to illustrate the typical phantom fabrication process. As mentioned above, anthropomorphic phantoms can be printed using FDM printers with modified parameter settings and materials. Some studies have shown that combinations of different materials, infill percentages, and infill patterns were able to replicate most tissue types including fat, soft tissue, lungs, and bone with acceptable dose distribution.^6^, ^26^, ^43^ Furthermore, Hounsfield Unit (HU) values of printed objects out of PLA and a few similar printing materials such as high impact polystyrene (HIPS) could be predicted using a quadratic best‐fit equation relating the HU values to the infill percentage $a{\left(I\%\right)}^{2}+b\left(I\%\right)=HU$, where *a* and *b* denote fitting coefficients, and *I%* denotes the infill percentage.^51^

**FIGURE 6:**
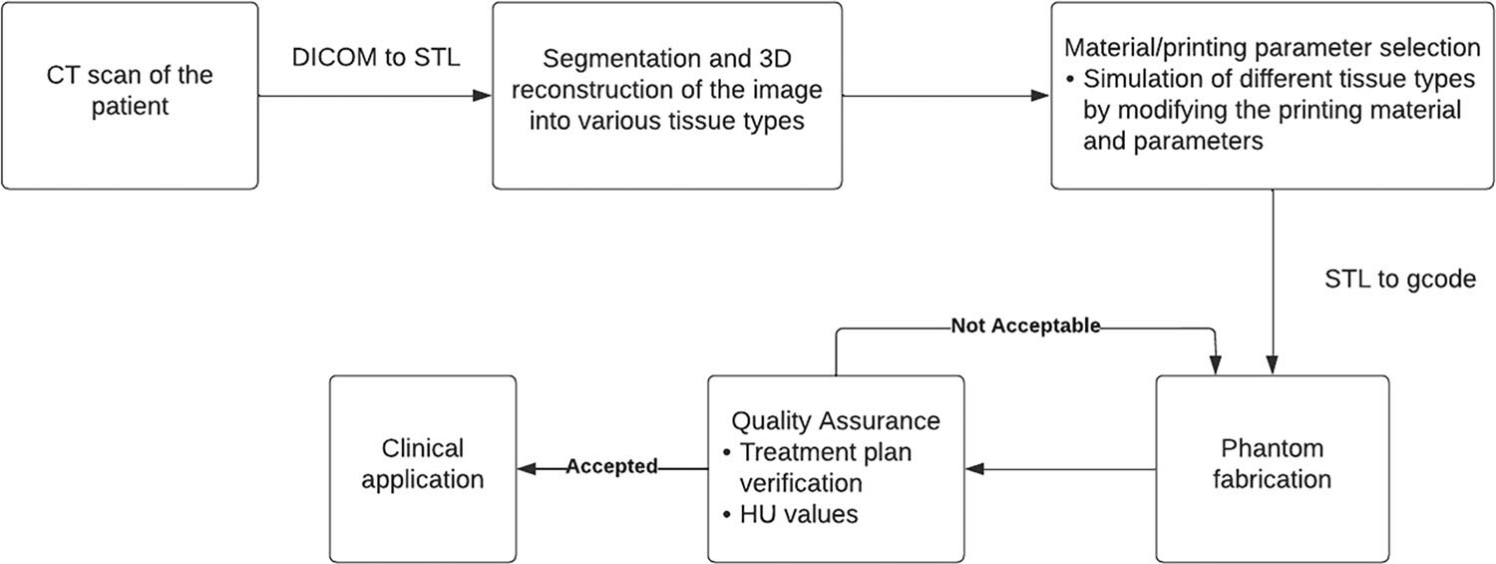
A typical workflow for fabrication of 3D printed anthropomorphic phantoms.

The typical workflow to print an anthropomorphic phantom is shown in Figure 6. Just like for printing other devices, a patient CT is obtained and imported into a TPS for the delineation of volume of interest (VOI). A DICOM analysis software such as OsiriX may replace the TPS. The images are imported into a DICOM analysis software for outline contouring and segmentation into various tissue types. The HU values of various tissue types within the desired VOI are measured and used as a reference for the selection of materials and printing parameters. The segmented images are then exported as STL to a 3D mesh processing software for final editing. Additional smoothing and cropping may be done if necessary during this step. For large phantoms, dividing the phantom into separate parts may be required due to limited printing capacity (i.e., maximum printable size). To this end, phantoms may be printed in multiple slices.^37^ In such cases, each slice must be examined for overhanging parts that could cause printing errors. Holes for immobilization rods may also need to be designed. After the necessary modification, the mesh is converted into gcode and printed out by varying the printing parameters to replicate different types of tissues.

Other simple phantoms, which are generally used for 3D printing verification purposes, such as material testing and calculation of optimal printer settings, are usually printed out in forms of a small slab, cylinder, or cube. Fabricating this type of phantom should be relatively straightforward compared to other devices; one can simply design the structure in CAD software (Fusion360, AutoCAD, etc.) and print it out by following the steps described in Section 2.1 (STL → gcode → printer).

## 4 QUALITY ASSURANCE

The data conversion and 3D printing processes contain risks of numerous systematic errors which may result in greater penalties in the later steps, requiring further QA procedures to minimize such errors. However, despite its necessity, there still exists no established set of QA guidelines for 3D printing processes or printed devices. There is a paucity in the exploration of QA for the 3D printing workflow or end products in radiation oncology, and only a few studies have investigated QA of 3D printed boluses.^18^, ^52^ Hence, this section attempts to provide examples and recommendations of QA procedures based on our own experience and these few studies. It is worth noting that some recommendations are specific to a given use case and might differ based on individual experience. Nevertheless, this section can hopefully fill the void and provide a quick guide for any departments utilizing 3D printing or considering its implementation, before the future advent of any relevant AAPM task group reports.

### 4.1 QA of the 3D printing system

Recommended QA procedures and frequency provided by printer manufacturers should be considered when establishing in‐house QA programs. We recommend printing benchmarking devices and performing a comprehensive assessment. Benchmarking devices can be objects with simple, known geometries, such as a cylinder, or can be more complex and application‐specific such as a bolus or a surface applicator on a phantom. These devices should be printed following the standard printing workflows established in the clinic. The assessment should encompass the following aspects.

#### Visual inspection

Printed devices should first be inspected for major print/structural defects or imperfections.

#### Geometric evaluation

The geometric accuracy of printed devices should be assessed. For objects with a simple shape, such as a cylinder, the dimensions and volume can be directly measured on the object using a ruler to verify the print scale. For more complex devices, the object may be volumetrically imaged using a CT scan and compared against the designed dimensions. To quantify the differences between the two shapes, Hausdorff distances are often measured using a 3D image analysis software such as 3DSlicer and Polyworks, calculating the mean, median, maximum, and minimum distances for evaluation. Additionally, surface areas and volumes under cross‐sectional profiles can also be computed and compared to further ensure geometrical accuracy. To quantify the volumetric similarity or difference between the two shapes, metrics such as the dice similarity coefficient can be used.

#### Air gap evaluation

Additionally, for printed boluses and applicators, inspection of the air gaps between the device surface and patient or phantom surface should be completed. Quantitative metrics described above, such as Hausdorff distances, may be applied for this purpose.

#### Density evaluation

The density should be inspected by acquiring CT images of the printed device and evaluating the HU values using a TPS or other suitable image analysis software. Basic HU statistics, including mean, median, maximum, minimum, and standard deviation, should be calculated for quantification. Threshold values should be set up to identify areas where HU values fall outside the expected window. Observed HU values can also be translated into corresponding mass density and electron density units to be compared with the expected values.

#### Dosimetric evaluation

Dosimetric evaluation should be carried out to analyze dosimetric endpoints using the printed bolus, applicator, or phantom. Dose can be compared in a TPS between calculations on the image set of the printed device and calculations on the original design, or against measurements. To analyze the treatment plan and dose distribution, various dosimetric parameters can be calculated and compared, such as mean dose, maximum dose, minimum dose, variation, *D_n_*_%_ (the dose that covers *n*% of the target volume), *V*_m%_ (the target volume that receives more than m% of the prescribed dose), and homogeneity index.

#### Catheter passage evaluation

For HDR applicators, catheter passage should be evaluated by running the HDR catheters as well as active/dummy source wires into the printed channels. A range of channel diameters and channel curvatures can be tested to optimize the channel diameter and an acceptable curvature range. Channel smoothness can also be evaluated through visual inspection or image analysis.

#### Catheter locking evaluation

For HDR applicators, the catheter locking mechanism should also be evaluated with the printed applicator to ensure the catheter is fixed in position and stays still during the treatment.

#### 4.1.1 Case‐specific QA

Case‐specific QA for each printed device should be performed to confirm print quality. Any device that will be used for patient treatment should be visually inspected and evaluated for geometric, density, and dosimetric integrity, as described above. The device should be labeled with patient information, orientation, and position relative to isocenter. Additionally, we recommend re‐simulating the patient with the bolus or applicator in place. At the re‐simulation, the bolus placement should be inspected for correct positioning, proper fit, and patient comfort. The placement on the patient should be secure. Scanned images should be analyzed to ensure minimal air gaps. A threshold should be established for in‐house air gap tolerance based on acceptable dosimetric uncertainty.^53^, ^54^, ^55^, ^56^, ^57^ The treatment plan, designed in the earlier stages of the bolus fabrication, must be verified prior to clinical use by performing a final dose calculation using the re‐simulated CT scan of the patient with the printed bolus in place. One study has explored the QA tolerance levels for 3D printed boluses for VMAT treatment of the nose, and recommended 5% for density variation, 1 mm for thickness variation, and 5 mm for air gap.^18^ Another study used shape, bulk density, and defect characteristics to QA 3D printed boluses.^52^

For brachytherapy surface applicators, catheter passage should be evaluated in addition for every printed device that will be used for patient treatment, as described above.

For printed multi‐slice phantoms, the geometric, density, and dosimetric accuracy of both the slices and the complete phantom (with immobilization rods placed to hold all slices together) should be verified.

#### 4.1.2 QA frequency

In our clinic, a comprehensive QA of the 3D printing system is performed semi‐annually by printing homogeneous cylinders of two sizes to evaluate the stability of the printing accuracy and speed. We then evaluate the dimensions, density, and dosimetric accuracy of the printed cylinders, as described above. This test is also performed following the acquisition of a new batch of printing material and after any printer repair.

Case‐specific QA is performed for every device used for patient treatment.

Whenever changes are made to the printing workflow, an end‐to‐end test of the system is performed. This includes scanning an anthropomorphic phantom, designing the bolus or applicator, printing the device, and applying the case‐specific QA steps described above.

## 5 ADDITIONAL COST DISCUSSION

Another important aspect to consider for 3D printing clinical applications is the cost. The implementation of 3D printing is impractical if the cost is unreasonably high. Cost was not always reported in the clinical and investigational literature. Fortunately, multiple studies have demonstrated that one of the main advantages of 3D printing is the relatively low capital and consumable cost.^6^, ^12^, ^21^, ^58^ We have summarized the reported data relating to the printing cost in Table 2. In one paper, the cost of a FDM printer was reported to be approximately $1000, with materials for a variable density phantom costing less than $100,^6^ though most studies appear to use printers that cost $1000‐$3000.

**TABLE 2:** Summary of cost reports in 3D printing applications in radiation oncology.

Device	Source	Material	Cost	Print time	Note
Bolus					
Breast	Park et al.^60^	PLA	–	6 h	Based on six patients
Breast	Park et al.^61^	PLA	$30/bolus	12 h	Able to make two 5 mm thick boluses with 1 kg of PLA
Chest wall	Hou et al.^62^	Silicone, PLA	–	8.5–12 h	
Chest wall	Robar et al.^63^	PLA	$10/bolus	7–28 h, median 10.8 h, mean 12.6 h	No irregularities detected during QA
Ear	Zou et al.^64^	PLA	Depend on the printing size	6–9 h	
Eye canthi	Lukowiak et al.^65^	PLA	–	0.5–5 h	
Head & neck	Chiu et al.^48^	Silicone	$7.75–$28.41 per material	4–6 h	Bolus volume varying from 98 to 557 mL; mold weight varying from 81 to 228 g
Nose	Albantow et al.^59^	PLA	$16.61 (low infill)–$17.98/23.84 (high infill)	8.82 (low)–12.45 min (high)	Low infill = 18%, high infill = 100%
Nose	Canters et al.^66^	PLA	$30/patient (including material, print, and labor cost)	5–10 (4‐h reduction compared to conventional method)	28 euros per patient; 5 from material cost, 13 from depreciation cost, rest from printer maintenance
Nose	Kim et al.^13^	ABS‐M30	–	3–4.5 h	
Nose, scalp	Burleson et al.^12^	ABS, PLA	<$3,000 per printer	4–6 h	
Scalp, nose, chin, ear	Park et al.^16^	Silicone	–	3–32 h	Mold casting method for silicone rubber (Dragon skin) and urethane liquid rubber (Clear Flex 30); direct printing method for ninjaflex
Scalp	Baltz et al.^67^	Agilus‐60	$2,381.50 per material and labor	40 h	
**Brachytherapy**					
Eye, nose	Arenas et al.^21^	PLA	$9–$11.11	6.25 h	Failed molds cost $1.2–1.5; each mold printed at 90% infill and weighing 94 g
Facial skin	Bieleda et al.^22^	PLA		8–15 h	
**Phantom**					
Sagittal‐sliced, Full‐scale Upper body	Craft et al.^37^	PLA	$524 in raw materials; <$15 000 in fixed cost (software, hardware, etc.), which is about 25% cheaper than the traditional method	267.5 h	11‐slice phantom, print time will be lower if multiple printers are used simultaneously
Chest, variable Density	Choi et al.^68^	PLA	–	19–50 h	Mold weight varying from 329 to 975 g
Torso, variable Density	Mille et al.^26^	PLA	$160 (material)	15–24 h for each block	Printed in multiple blocks, total weight 2.4 kg
Femur bone	Tino et al.^69^	PLA	$10 per phantom	10 h	30 g of Fe‐PLA and 130 g of standard PLA
Prostate	Chiu et al.^68^	Silicone, PVC	$23.98 per phantom	1.5–2 h	Coflex 00−30 silicone, emulsion silicone with 20% or 50% mineral oil, and regular or supersoft polyvinyl chloride (PVC). Commercial prostate phantom costs $300–$500.

According to one study, 3D printing can reduce the HDR applicator production time by 34% (from 211.5 to 152.5 h) and the financial cost by nearly 50%, compared to casting techniques.^21^ Another study found that the average staff time and material cost of a 3D‐printed bolus were $10.58 and $13.87, respectively. These numbers are significantly smaller than those of wax bolus, which were $138.54 and $20.49, respectively, due to less staff training and the printer not requiring active monitoring.^59^ However, it should be noted that the staff time cost may be significantly higher than their reported data depending on the complexity of printed product and considering the time spent on the extra simulation scan, image processing, and post‐print device processing.

As described in an earlier section, much of the software necessary for data conversion and processing is freely available. Other software options that are not free may increase the short‐term cost, but the effect may be negligible in the long term if the software is easier to use. Furthermore, printer settings, including infill density, infill pattern, and print speed, may also influence the print cost by affecting print time and material usage,^39^ though the effect from a small change may be negligible.^36^ Hence the print time must be estimated and taken into consideration during the design and planning stages.

In addition to in‐house printing, vendors like Adaptiiv and .Decimal (dotdecimal.com) also provide mail‐order services for customers without a 3D printer or customers that desire a higher‐quality bolus that their in‐house system could produce. Digital files in STL format are exported by the customer and securely sent to the vendor for printing. Within 3–5 days, the vendor will ship the printed bolus along with a Certificate of Analysis documenting any QA performed. Using this option gives customers access to state‐of‐the‐art printers and versatile print materials such as the semi‐flexible TPU and highly flexible silicone, while reducing personnel time associated with the print. Print costs are usually several hundred US dollars per print, and may vary based on print volume, print material, and use of vendor‐specific software.

## 6 CONCLUSION AND OUTLOOK

This paper has attempted to provide guidance on clinical implementation of additive manufacturing, investigating topics in fabrication process, QA, and cost analysis. The advantages of 3D‐printed tools are clear, and they have an immense potential to advance the quality of radiotherapy in the near future. An increasing number of clinical studies and review papers have demonstrated improved outcomes in many areas through 3D printing, including cost efficiency, dosimetric measurements, and physical accuracy. Undoubtedly, the 3D printing industry will keep experiencing rapid advancements to make the technology even more accessible and precise. However, there still exists certain problems that remain unsolved. Two major problems are material choice and optimal printing parameters. PLA and ABS are suitable materials for boluses and applicators, but numerous studies suggest that there are opportunities for improvement, reporting that the development of new bolus material is necessary and urgent. Furthermore, only a very few studies have experimented with different sets of printing parameters; future studies must also focus on establishing a set of ideal printing parameters for each type of device and printer to maximize the cost efficiency and utility of 3D printing in radiation oncology. Furthermore, to facilitate the 3D printing utilization in radiation oncology, it would be desirable to integrate STL design and import/export directly into TPS. At the same time, universal design standards also need to be established. This work provides a first step towards defining practice guidelines for the increasingly important practice of 3D printed devices and applications in radiation oncology.

## AUTHOR CONTRIBUTIONS

Michael Ashenafi: Conceptualization, methodology, investigation, writing original draft. Seungkyo Jeong: Methodology, investigation, data collection and analysis, writing original draft. Joshua N. Wancura: Methodology, writing original draft. Lang Gou: Methodology, data collection and analysis. Matthew J. Webster: Methodology. Dandan Zheng: Conceptualization, methodology, investigation, data collection and analysis, writing original draft, supervision.

All authors contributed to review & editing of the manuscript.

## CONFLICT OF INTEREST STATEMENT

The authors have no relevant conflicts of interest to report.
